# MAV_4644 Interaction with the Host Cathepsin Z Protects *Mycobacterium avium* subsp. *hominissuis* from Rapid Macrophage Killing

**DOI:** 10.3390/microorganisms7050144

**Published:** 2019-05-21

**Authors:** Matthew S. Lewis, Lia Danelishvili, Sasha J. Rose, Luiz E. Bermudez

**Affiliations:** 1 Department of Biomedical Sciences, College of Veterinary Medicine, Oregon State University, Corvallis, OR 97331, USA; Matthew.lewis@oregonstate.edu (M.S.L.); lia.danelishvili@oregonstate.edu (L.D.); sasha.rose@oregonstate.edu (S.J.R.); 2 Department of Microbiology, College of Science, Oregon State University, Corvallis, OR 97331, USA

**Keywords:** *Mycobacterium avium* subsp. *hominissuis*, cathepsin Z, macrophage, nitric oxide, ADP-ribosyltransferase, bacterial killing

## Abstract

*Mycobacterium avium* subspecies *hominissuis* (MAH) is an opportunistic pathogen that is ubiquitous in the environment and often isolated from faucets and showerheads. MAH mostly infects humans with an underlying disease, such as chronic pulmonary disorder, cystic fibrosis, or individuals that are immunocompromised. In recent years, MAH infections in patients without concurrent disease are increasing in prevalence as well. This pathogen is resistant to many antibiotics due to the impermeability of its envelope and due to the phenotypic resistance established within the host macrophages, making difficult to treat MAH infections. By screening a MAH transposon library for mutants that are susceptible to killing by reactive nitrogen intermediaries, we identified the *MAV*_4644 (*MAV*_4644:Tn) gene knockout clone that was also significantly attenuated in growth within the host macrophages. Complementation of the mutant restored the wild-type phenotype. The *MAV*_4644 gene encodes a dual-function protein with a putative pore-forming function and ADP-ribosyltransferase activity. Protein binding assay suggests that MAV_4644 interacts with the host lysosomal peptidase cathepsin Z (CTSZ), a key regulator of the cell signaling and inflammation. Pathogenic mycobacteria have been shown to suppress the action of many cathepsins to establish their intracellular niche. Our results demonstrate that knocking-down the cathepsin Z in human macrophages rescues the attenuated phenotype of *MAV*_4644:Tn clone. Although, the purified cathepsin Z by itself does not have any killing effect on MAH, it contributes to bacterial killing in the presence of the nitric oxide (NO). Our data suggest that the cathepsin Z is involved in early macrophage killing of MAH, and the virulence factor MAV_4644 protects the pathogen from this process.

## 1. Introduction

*Mycobacterium avium* subspecies *hominissuis* (MAH) is a ubiquitous environmental saprophyte capable of infecting a broad host range of animals, including humans [1]. MAH creates robust biofilms and is often isolated from outputs of modern chlorinated drinking water distribution systems, such as showerheads and faucets [2,3]. While MAH mainly infects immunocompromised individuals and patients with an underlying lung disease, such as chronic obstructive pulmonary disorder, cystic fibrosis, it is now evident that MAH can also cause progressive lung infections in patients without underlying lung disease [4]. The gut is also a route of MAH entry, and the increasing prevalence of MAH is apparent in both respiratory and gastrointestinal infections [5,6,7]. Patients with MAH infections are typically given a combination therapy with two or three antimicrobial agents for one year, increasing chances of development of drug-resistance. Among many factors including the bacterial intrinsic resistance makes MAH infection difficult to treat [8]. This intrinsic resistance in part is attributed to the impermeability of the mycobacterial envelope, which is rich in hydrophobic mycolic acids [9]. In addition, MAH resides and replicates within the host macrophages, further isolating the bacteria from effective levels of circulating antibiotic [10].

Overcoming the bactericidal properties of macrophage is a key factor for successful survival of intracellular pathogens such as mycobacteria [11]. One principal mode of circumventing the MAH killing by macrophages is to arrest the phagosome maturation. Following phagocytosis, MAH resides in a non-acidified phagosome, denoted the mycobacteria containing phagosome (MCP) [2]. The MCP fuses with early endosomes, making MAH phagosome accessible to some endosomal proteins; however, the pathogen restricts accumulation of proton-ATPases, halting acidification [12]. The MCP also co-localizes with cathepsin D, a mediator in apoptosis and in antigen presentation mechanisms as an immature proenzyme [12,13]. Cathepsins play a major role as lysozyme proteases as well as in cell signaling and inflammation, and their importance to control mycobacterial infections has been shown [14]. By manipulating the function of cathepsins, the infecting organism is able to successfully establish the intracellular niche in phagocytic cells. In addition, pathogenic mycobacteria secrete at least two highly conserved superoxide dismutase enzymes, which confer protection from oxidative stress and this is required for full virulence [15,16,17].

Virulence factors of pathogenic mycobacteria are either secreted or expressed on the bacterial outer cell envelope [18]. In addition to the housekeeping Sec and Tat transport systems, mycobacteria utilize a type VII secretion (T7S) system to secrete proteins [19]. T7S systems of mycobacteria are encoded by 5 separate *esx* gene clusters, denoted ESX 1-5 [20]. ESX1 required for *Mycobacterium tuberculosis* virulence secretes ESAT-6 protein, which open pores in the MCP envelope allowing the pathogen access the host cytosol [21]. Interestingly, two species of virulent mycobacteria, *M. ulcerans* and MAH, have lost ESX1 to a deletion event [19,22]. While *M. ulcerans* retains virulence by secreting a plasma-encoded polyketide toxin, causing macrophage cytotoxicity [23], no toxins have been described in MAH.

To identify the virulent-related genes of MAH associated with the resistance to oxidative killing mechanism of macrophages, we screened a transposon library and identified the MAV_4644 gene knockout clone susceptible to nitric oxide. MAV_4644 was computationally identified as a putative pore-forming protein with ADP-ribosyltransferase (ADPRT) activities [24]. ADPRT proteins comprise a family of toxins which add ADP-ribose from NAD to host proteins, halting key cellular processes. Its known that Cholera and pertussis toxins, responsible for thousands of annual deaths, are members of the ADPRT family [25]. In this study, we focused on functionally characterizing the MAV_4644 protein that could potentially act as a pore and a toxin, and discovered the novel virulence mechanism of MAH.

## 2. Materials and Methods

### 2.1. Bacterial Strain

*Mycobacterium avium* subsp. *hominissuis* strain 104 (MAC104) was originally isolated from the blood of an AIDS patient and has been shown to be virulent in mice infected by IV or respiratory routes. MAC104 were cultured on Middlebrook 7H10 agar supplemented with 10% oleic acid, albumin, dextrose and catalase (OADC) (Hardy Diagnostics, Santa Maria, CA, USA) to mid log-phase at 37 °C. Growth curves were conducted in Middlebrook 7H9 broth with 10% OADC and with and without glycerol supplement. The optical density at 600 nm was periodically measured with a spectrophotometer.

### 2.2. Host Cells

The human monocyte THP-1 cell line (ATCC, Manassas, VA, USA) was cultured in RPMI-1640 (Corning, Teksbury, MA, USA) supplemented with 10% fetal bovine serum (FBS, Gemini, Bridgewater, OR, USA) at 37 °C with 5% CO_2_ as suspension cells. Cells were counted with a hemocytometer, seeded at 80% confluency into 48-well plates and supplemented with 20 ng/mL of phorbol 12-myrisate 13-acetate (PMA, Sigma Aldrich, St Louis, MO, USA) to differentiate into adherent macrophages [26]. After 24 h, the culture media were replaced and cells were allowed to rest an additional 24 h before infection assays. The murine RAW 264.7 cell line (ATCC) was cultured in DMEM (Corning) supplemented with 10% FBS at 37 °C with 5% CO2. RAW264.7 macrophages were treated for 10 min at 37 °C with 5 mM EDTA in phosphate buffered saline (PBS) and harvested by gentle washing with a serological pipette. Cells were pelleted by centrifugation to remove the EDTA, resuspended in fresh culture media and counted with a hemocytometer to seed at 80% confluence at the time of infection.

### 2.3. MycoMarT7 Transposon Library Screening for Nitric Oxide Susceptibility

MycoMarT7 (Mmt7) is a phagemid with a kanamycin resistance gene under the control of T7 promoter [27]. MAC104 was transduced with MmT7 as previously described [28]. A total of 930 MAH Mmt7 transposon mutant (Mmt7:Tn) individual colonies were each grown in 96-deep-well plates for 5 days in 300 µL of 7H9 broth supplemented with 10% OADC (Hardy Diagnostics) and 400 µg/mL of kanamycin (Sigma) at 37 °C with 210 rpm shaking. Two wells in each plate were inoculated with MAC104 without kanamycin as a positive control and one well filled with just media alone served as a blank. After 5 days of growth, 150 µL of bacterial cultures were transferred to 96-well flat bottom plates and pelleted by centrifugation. The supernatant was removed and bacteria were carefully resuspended in fresh 7H9 broth supplemented with 10% OADC, 400 μg/mL of kanamycin and 100 µmol of spermine NONOate, a nitric oxide donor (Calbiochem, San Mateo, CA, USA. The optical density was measured at 600 nm in a plate-reading spectrophotometer (Biotek, Shoreline, WA, USA) and recorded as time 0. The plates were placed at 37 °C with 210 rpm shaking for 3 h, then moved to a 37 °C incubator for an additional 2 days. The optical density was read at 600 nm and the percent of bacterial increase was determined. Mutants with increased susceptibility to nitric oxide were identified as those with 50% or less optical density when compared to the wild type. Identified mutants were cherry-picked from the library and results were confirmed by repeating the assay.

### 2.4. Sequencing of Mmt7 Gene Knockout Clones

Mmt7 transposon insertion sites were identified via ligation-mediated PCR (LMPCR) as previously reported [28]. Briefly, genomic DNA was isolated, digested with restriction enzyme, and ligated with custom adapters complementary to the cut sites. Resulting products were used as templates for the LMPCR reaction with primer sets designed to amplify a 150-bp segment of the transposon and the region of interrupted gene at the insertion site. Amplicons were submitted to the Center for Genome Research and Biocomputing at Oregon State University for Sanger sequencing.

### 2.5. Complementation of MAV_4644 Gene Knockout Clone

The 2478-bp coding fragment of *MAV*_4644 was PCR amplified from the genomic DNA of *M. avium* 104 strain, and cloned into HindIII and NotI restriction sites of the mycobacterium chromosomal integration vector pMV306 containing the apramycin resistance marker. The resulting vector was electroporated into MAV_4644 gene deficient transposon clone, and *M. avium* transformants were plated on 7H10 Middlebrook agar plates containing 400 µg/mL of apramycin. The positive *M. avium* complemented clone was identified by PCR using MAV_4644 gene-specific primers, where *MAV*_4644 gene knockout clone served as a negative control.

### 2.6. Uptake and Survival Assays

A single cell suspension of the wild-type MAH, *MAV*_4644:Tn mutant and complemented clone was created in phosphate buffered saline (PBS) to adjust to a McFarland standard #2 (3 × 10^8^ cells/mL). Inoculums were also plated for the colony forming unit (CFU) counts. The bacterial suspension was left still for 5 min to allow bacterial aggregates to settle and the top half volume was used for infection assays. Host cells were seeded in either 24-well or 48-well flat bottom plates and inoculated at a multiplicity of infection of 10 bacteria to 1 host cell, and centrifuged for 10 min at 800 rpm to synchronize the infections. The chasing was allowed to proceed for 2 h at 37 °C and 5% CO_2_. Infected cells were then gently washed twice with HBSS and refreshed with the appropriate culture medium supplemented with 200 µg/mL of gentamicin (Sigma) for 2 h to eliminate extracellular bacteria. The cells were then washed twice with HBSS (Corning). At this point, the wells denoted time 0 were lysed by adding 0.1% Triton X-100 (VWR) for 10 minutes and disrupted with twenty pipette strokes, while the remaining wells were replenished with culture medium supplemented with 10 ng/mL of murine IFN-γ (Invitrogen) for collection at subsequent time-points. Cell lysates were serially diluted in PBS and plated onto Middlebrook 7H10 agar supplemented with 10% OADC (Hardy Diagnostics). Colonies were counted after ten days of incubation at 37 °C and results were compared between the control (wild-type MAH) and MAV_4644 gene knockout and complemented strains.

### 2.7. In Silico Analysis

The National Center for Biotechnology (NCBI) web server was utilized for conserved protein domain searches. The NCBI Basic Local Alignment Search Tool (BLAST) was utilized for nucleotide and protein alignment analysis. A search for homologous proteins was performed on the Kyoto Encyclopedia of Genes and Genomes (KEGG) web server using the Sequence Similarity Database (SSDB), which ranks all possible pairwise protein alignments based on the Smith–Waterman similarity score. The dendogram was created based on the total scores [29]. The Constraint-Based Local Alignment Tool (COBALT) was utilized to visualize primary protein sequence alignments [30]. The association of genes in an operon was investigated on the Database of Prokaryotic Operons (DOOR2) web server [31].

### 2.8. Expression of MAV_4644, MAV_4643, and MAV_4642 Recombinant Proteins and Immunoprecipitation Assays

The C-terminal sequence of *MAV*_4644 corresponding to the VIP2 ADP-ribosyltransferase domain, denoted MAV_4644_CTD, as well as *MAV*_4643 and *MAV*_4642 were cloned into the pET6xHN-C vector (Clontech, San Francisco, CA, USA). The recombinant protein products were expressed in BL21 (DE3) competent *E. coli* (Invitrogen, Calsbed, CA, USA) and purified with the His-60 Ni Superflow Gravity Column Purification Kit (Takara, Salem, OR, USA following the manufacturer’s recommendations. The expression and purification of the recombinant proteins were confirmed by SDS_PAGE and Western blot using an anti-6xHN antibody (Takara).

For the immunoprecipitation assay, the recombinant *MAV*_4644_CTD, *MAV*_4643 and *MAV*_4642 protein purifications were arrested before elution. The cleared THP-1 macrophage lysate was added to the columns, and incubated at 4 °C for 24 h with gentle inversion. The next day, columns were drained, washed with 5 mL of equilibration buffer and then 5 mL of wash buffer. The host bound proteins to the recombinant protein were eluted into 1-mL fractions with elution buffer. The total protein concentration in each fraction was assessed by SDS-PAGE and Coomassie staining. The second fraction was identified to contain the highest concentration of total protein in each pull-down assay. These fractions were concentrated to 50 µL using a Nanosep 3K centrifugal filter device (VWR) and sequenced at the Oregon State University Mass Spectrometry Center following the standard protocols. Protein samples were Proteome Discoverer v 1.3.0, Mascot v 2.3 and Scaffold were used for data analysis. The Uniprot *Homo sapien* database was used to identify host proteins. A lysate of un-induced *E. coli* DE3 was used as a negative control in place of the recombinant MAH proteins. The proteins identified in the negative sample were subtracted and the CRAPome database was used to filter out nonspecific interactions [32].

### 2.9. The siRNA Knock-Down of Cathepsin Z

THP-1 cells were seeded to 60% confluency in 48-well plates and differentiated with 20 ng/mL of PMA in RPMI-1640 (Corning) supplemented with 10% FBS (Gemini, New York, NY, USA) for 24 h, at which point the medium was replenished. Cells were rested an additional 24 h. The culture medium was refreshed again just before adding the freshly prepared transfection complex. The transfection complex was prepared with either cathepsin Z interfering RNA designed at Custom Dicer-Substrate siRNA (Integrative DNA Technologies, IDT, Coralville, IA, USA) or non-targeting interfering RNA (IDT) and Viromer^®^ Green transfection reagent (Lipocalyx, Houston, TX, USA) following the manufacturer’s protocol and with the following specifications: the working volume per well was 500 mL and each transfection complex was made at 100 nM siRNA in 50 µL of Viromer Green buffer. The uptake of transfection complex was verified with the TYE 563 Transfection Control (IDT). The transfection medium was replaced at 24 h with RPMI-1640 supplemented with 10% FBS (Gemini) and refreshed in 24 h intervals for 3 days. Cells were then infected with the wild-type MAC104 and MAV_4644:Tn. At each time points, cells were lysed to examine bacterial survival and assess CTSZ protein levels. The cell lysates were resolved with SDS-PAGE, transferred to a nitrocellulose membrane, probed with cathepsin Z (ThermoFisher Scientific, San Jose, CA, USA) and B-actin (Abcam, Boston, MA, USA) antibodies and visualized with a LI-COR imaging system (Lincoln, NE, USA).

### 2.10. Cathepsin Z Activity against MAC104 and MAV_4644:Tn

To determine whether purified cathepsin Z was able to kill MAC104, in vitro culture of MAC104 or MAV_4644:Tn at concentrations of 10^5^, 10^4^, and 10^3^/mL were incubated with cathepsin Z (0.5 M and 2 M; ThermoFisher) for 30 min and CFUs/mL of viable bacteria were determined on 7H10 agar plates. In subsequent experiments, cathepsin Z was added to the bacteria culture in the presence of 100 µmol of spermine NONOate, as described above, and bacterial CFUs were determined at the same time points.

### 2.11. Statistical Analysis

Experiments were repeated at least 2 times. Data is displayed as the means of replicates ± standard error. The Student’s *t*-test, ANOVA and Mann–Whitney tests were used to compare experimental and control groups. Differences were considered significant when *p* < 0.05. The Holm–Sidak approach (GraphPad Prism version 7.0 software, San Diego, CA, USA) was used for multiple comparisons.

## 3. Results

### 3.1. Nitric Oxide Susceptibility of MAH

**Figure 1:**
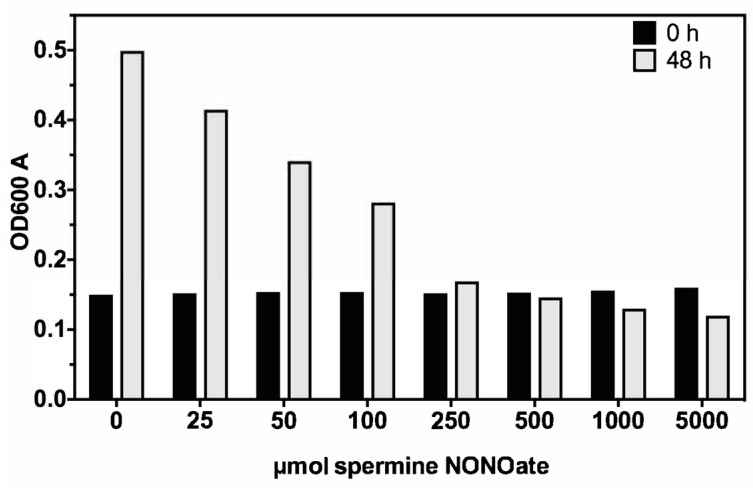
In vitro susceptibility of MAC104 to spermine NONOate. MAC104 was grown to OD600 of 0.15 in 7H9 broth supplemented with 10% oleic acid, albumin, dextrose and catalase (OADC) in a 37 °C shaker. Spermine NONOate, a nitric oxide donor, was added at reported amounts and the cultures were incubated for an additional 48 h. The data is representative of three separate trials.

A dose response effect of MAC104 growth inhibition was investigated for a 0 to 5000 μmol concentration range of the spermine NONOate (Figure 1). While we found that 250–5000 μmol spermine NONOate similarly inhibited the bacterial growth during 48 h of incubation, 100 μmol concentration was near the threshold of sensitivity and, thus, this concentration was used in the transposon library screen to isolate nitric oxide sensitive mutants.

### 3.2. Isolation of MAH Mmt7 Transposon Mutants Susceptible to Low Bacteriostatic Concentration of Nitric Oxide

**Figure 2:**
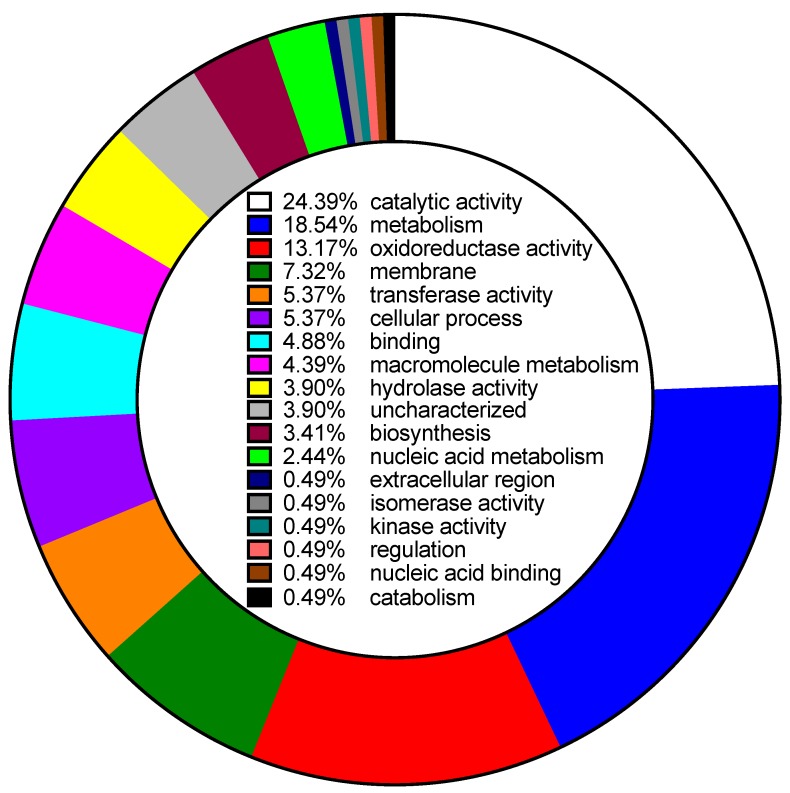
Gene ontology analysis of MAH Mmt7 transposon mutants identified in spermine NONOate susceptibility screen.

To identify the molecular mechanisms of nitric oxide resistance, 930 individual transposon mutants of MAC104 were screened for susceptibility to a sub-bacteriostatic level of nitric oxide. A total of 46 mutants displayed increased susceptibility when compared to the wild-type control. The transposon insertion site was determined to be located within a putative coding sequence in 34 of the susceptible mutants (Table 1). Gene ontology analysis, shown in the Figure 2, identified 24% involved in catalytic activity, 18% in metabolism, 13% in oxidoreductase activity and 7% part of the membrane.

### 3.3. MAV_4644:Tn Is Attenuated in Murine Macrophages

**Figure 3:**
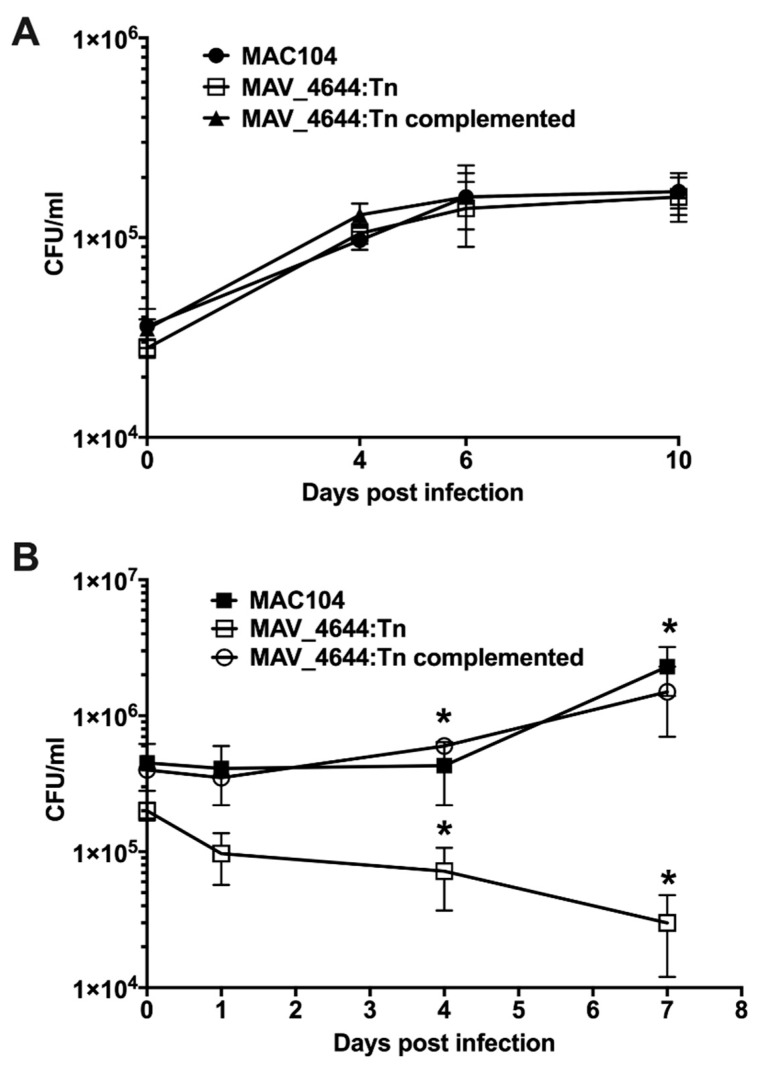
Inactivation of MAV_4644 gene. (**A**) Comparison of in vitro growth of wild-type MAC104, MAV_4644 gene knockout and complemented strain in the aerated 7H9 medium supplemented with OADC. (**B**) Survival of MAC104 (black squares), MAV_4644:Tn (open squares) and MAV_4644:Tn complemented clone (open circles) in murine macrophages. RAW264.7 cells were cultured in DMEM supplemented with 10% FBS in 24-well plates and infected with either the wild-type MAC104 or MAV_4644:Tn mutant for 2 h at 37 °C and 5% CO_2_. The cells were washed twice with HBSS and incubated for an additional 2 h in culture medium supplemented 200 µg/mL of gentamicin. Monolayers were washed and supplemented with 10 ng/mL of murine IFN-γ in DMEM. At reported time points, macrophages were lysed with 0.1% Triton X-100, serially diluted, plated on 7H10 agar plates and incubated at 37 °C for 8–10 days before colonies were counted for CFU. Survival results of the wild-type and complemented strain versus mutant were analyzed using Student’s *t* test. *, *p* < 0.05 was determined significant.

Because murine macrophages respond to Interferon gamma (IFNγ) by producing NO without log of time, we decided to investigate the intracellular growth phenotype using Raw 264.7 macrophages. Thirty-four nitric oxide susceptible transposon clones were tested for survival in an activated murine macrophage infection model for 7 days, and *MAV*_4644:Tn mutant was selected to be significantly deficient to grow within RAW264.7 cells. While in vitro studies revealed no difference between growth of wild-type MAC104, *MAV*_4644 gene knockout and complemented strains in the liquid culture medium (Figure 3A); the *MAV*_4644:Tn mutant was attenuated with approximately 1.5 log to grow within IFN-γ stimulated RAW264.7 macrophages when compared to the wild-type MAC104 infection (Figure 3B). Complementation of the *MAV*_4644 gene abolished the defect, and the clone grew in macrophages similar to the wild-type bacteria.

### 3.4. In Silico Analysis of MAV_4644

MAV_4644 is a dual-function protein with putative pore-forming and ADP-ribosyltransferase activities (NCBI Ref.Seq. WP_011726171.1). The conserved domain analysis of MAV_4644 protein in the Table 2 displays a WXG100 motif, a membrane translocation signal for type VII secretion systems [33], at the N-terminus of the protein (7 through 92 amino acids). Amino acid region 97 to 439 is predicted to be α-helical with putative pore-forming activity [24]. We also identified is a proline-rich disordered region within 455–655 amino acid sequence. The amino acid sequence at 675–822 corresponds to VIP2, a protein family of actin-ADP-ribosylating toxins. A protein similarity search, performed on the Kyoto Encyclopedia of Genes and Genomes (KEGG, 30) recognized the top ranked homologs as putative ADP-ribosyltransferases, conserved hypothetical proteins or putative alanine and proline rich proteins.

**Figure 4:**
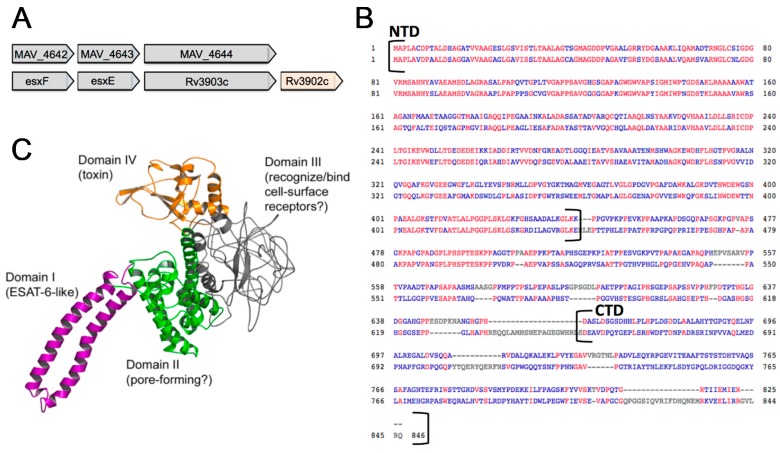
(**A**) Chromosome region of MAV_4644 operon in MAH and corresponding location in *M. tuberculosis*. (**B**) MAV_4644 and Rv3903c protein alignment. The N-terminal domain (NTD) has 100% query coverage with an E-value of 1E-166 and 73% GC content. CTD has no significant alignment with 52% GC content of gene in MAH and 68% in *M. tuberculosis*. (**C**) The I-Tasser predictive model of MAV_4644 showing four.

MAV_4644 resides in a three-gene operon with MAV_4642 and MAV_4643 (Figure 4A). MAV_4642 protein (GenBank: ABK64648.1) and MAV_4643 protein (GenBank: ABK68102.1) are annotated as conserved hypothetical secretion proteins and they both contain the WXG100 motif, characteristic for type VII secretion targets [33]. A similarity search on KEGG-identified *M. tuberculosis* ESAT-6-like esxF and esxE proteins as homologues to MAV_4642 and MAV_4643 respectively. A comparison of the complete operons between two strains reveals syntenic conservation of gene order, though the immunizing factor encoded by Rv3902 is lacking in MAH [34].

MAV_4644 homologue protein in *M. tuberculosis* was identified as Rv3903c (Figure 4A,B). This protein is well characterized as a channel protein with necrosis-inducing toxin function (CpnT) [35]. The N-terminal domain of CpnT (NTD: amino acid 1–443) is an outer membrane channel protein required for the uptake of glycerol. The C-terminal domain (amino acids 720–846) is released from the cell surface by proteolytic cleavage [35]. The released toxin, denoted TNT hydrolyzes NAD+, completely depleting this essential coenzyme and leading to necrotic cell death of the infected host cell [34]. Figure 4B shows the protein BLAST (pBLAST) alignment on the National Center for Biotechnology (NCBI) web server of the N-terminal domains CpnT and MAV_4644 protein along amino acid 1 to 435. Both proteins yield 100% query coverage with an E-value of 1 × 10^−166^. The pBLAST alignment of the whole proteins as well as the disordered region also shows considerable conservation through amino acid 546. However, the C-terminal regions of both proteins have divergent primary sequences, as pBLAST was only able to align 10% of these domains (Figure 4B). Using the Iterative Threading ASSEmbly Refinement (I-TASSER) software [36], the predictive model of MAV_4644 has been generated based on the protein structure and function shown in the Figure 4C.

The assay was performed as described in the materials and methods. MAH was grown in 7H9 broth with OADC. Spermine NONOate (100 µmol) was used as a source of NO. The data represent the findings of two independent experiments, at 30 min of bacterial incubation with cathepsin Z and/or NO. * *p* < 0.05, when MAV_4644:Tn clone compared with the wild-type MAC104 CFU counts.

### 3.5. Complementation of the MAV_4644:Tn Mutant Restores the Bacterial Survivability Phenotype

To determine whether the complementation of The MAV_4644:Tn would recover the function of the wild-type bacteria, we inserted the functional MAV_4644 gene into the chromosome of MAH mutant and examined intracellular bacterial growth within murine macrophages in comparison with the wild-type bacteria as well as the MAV_4644:Tn mutant growth. As shown in the Table 3, the MAV_4644:Tn complemented clone was able to survive in macrophages in a similar way as the wild-type bacteria recorded by intracellular bacterial colony forming units.

### 3.6. MAV_4644 Is Not Required for Growth in Defined Medium

**Figure 5:**
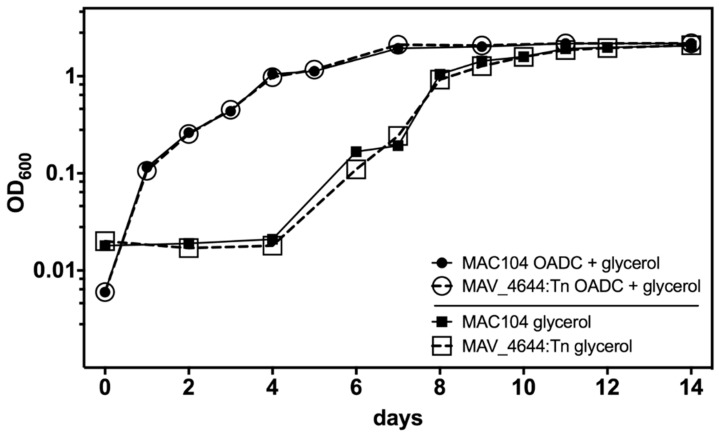
The growth kinetics of MAC104 and MAV_4644:Tn are similar whether assayed in 7H9 broth supplemented with 10% OADC and glycerol, or 7H9 supplemented with glycerol alone. The data is representative of three separate trials.

To determine whether the observed attenuation of *MAV*_4644:Tn in murine macrophages can be attributed to differences in growth rate, growth curves were ran in rich media. MAV_4644:Tn displayed the same growth kinetics as MAC104 in 7H9 broth supplemented with 10% OADC (Figure 5). Furthermore, to investigate whether MAV_4644 protein is required for MAH growth on glycerol, similar to CpnT in *M. tuberculosis* H37Rv, 7H9 broth was prepared without OADC, which removes the dextrose and leaves only glycerol as an energy and carbon source. The comparative growth curves ran in this media revealed that MAV_4644 is not required for the uptake of glycerol (Figure 5).

### 3.7. MAV_4644_CTD, MAV_4643, and MAV_4642 Recombinant Proteins Interact with the Cathepsin Z

Due to the nearly identical N-terminal regions of MAV_4644 protein and CpnT, we hypothesized that the CTD of MAV_4644 also undergoes proteolytic cleavage before secretion or is present on the outer membrane of MAH and is capable to interact with host proteins [35]. The MAV_4643 and MAV_4642 proteins, highly similar to esat6-like secreted proteins of *M. tuberculosis*, are most likely exported by the oligimerized MAV_4644 NTD pore [34].

To investigate potential host-binding partners for MAV_4644, MAV_4643, and MAV_4642 proteins, immunoprecipitation assays were performed with THP-1 clarified lysates. The MAV_4644 protein C-terminal region, corresponding to the VIP2 domain, was expressed and denoted MAV_4644_CTD. The pull-down assay with MAV_4644_CTD displayed interaction with three host proteins (Supplemental Table S1). The most noteworthy is the lysosomal peptidase cathepsin Z, which was also identified in pull-down assays with MAV_4643 and MAV_4642 proteins. In addition, six additional host proteins, including complement component 1 and cadherin 4, were identified during MAV_4643 interaction assay, whereas, MAV_4642 resulted in pulling 145 host proteins, including cathepsin S and beta-2-microglubulin (Supplemental Table S1).

### 3.8. MAV_4644:Tn Is Rapidly Killed in THP-1 Macrophages

**Figure 6:**
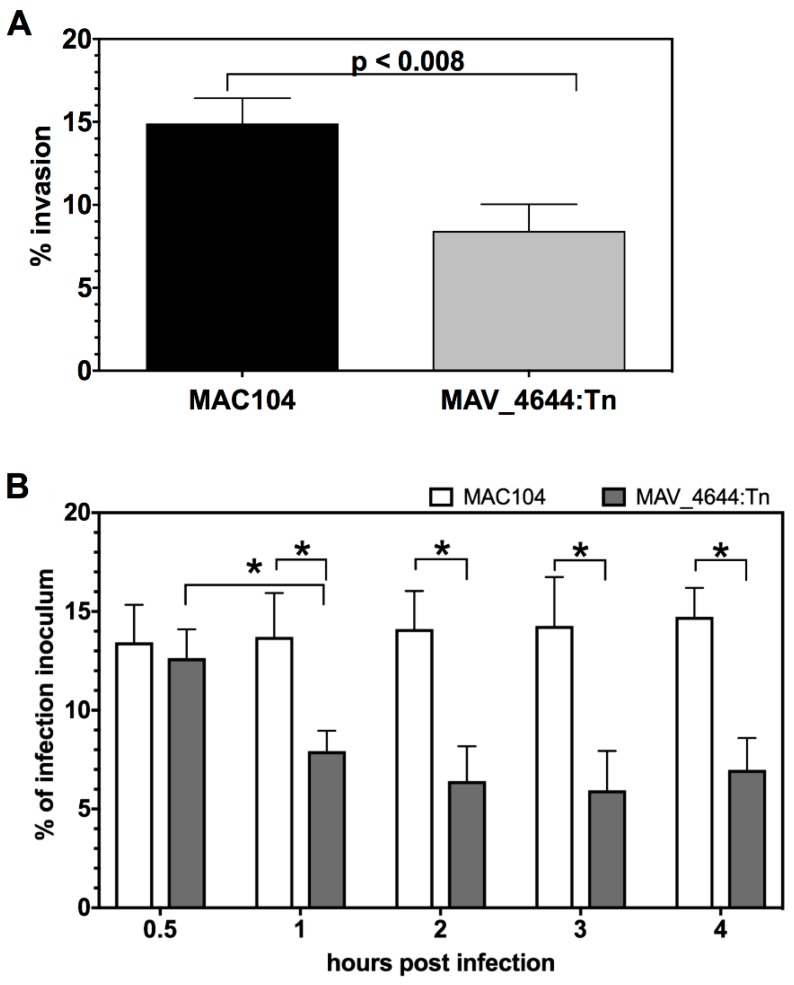
(**A**) The percentage of invasion of the wild-type MAC104 and MAV_4644:Tn clone. RAW264.7 cells were cultured in DMEM supplemented with 10% FBS in 24-well plates and infected with either the wild-type MAC104 or MAV_4644:Tn mutant for 2 h at 37 °C and 5% CO_2_. The cells were washed twice with HBSS, incubated for an additional 2 h in culture medium supplemented 200 µg/mL of gentamicin, and then lysed with 0.1% Triton X-100. The present of uptake was calculated by dividing cfu/mL at time 0 to the initial inoculum and multiplying by 100. The Student’s *t*-test was used to determine *p*-values as reported. (**B**) The percentage of MAC104 (white bars) and MAV_4644:Tn (grey bars) survival within 4 h of THP-1 macrophages infection. THP-1 macrophages were cultured in 48-well plates as describe above, and then infected with MAC104 or MAV_4644:Tn at an MOI of 10 for 30 min at 37 °C and 5% CO_2_. All monolayers were washed twice with HBSS and selected wells were lysed at 30 min for time 0, while RPMI-1640 supplemented with 10% FBS was replaced in the rest of the wells for subsequent time points. Cells were lysed with 0.1% Triton X-100, serially diluted, plated on 7H10 agar plates and CFU counts were recorded. Survival of the wild-type versus mutant was analyzed using Student’s *t* test. *, *p* < 0.05 was determined significant.

Due to the fact that cathepsins are proteolytic enzymes of lysosomes that function in the endocytic pathway and directly contribute to the pathogen killing during very early stages of mycobacterial infection, we investigated in more depth the differences in the intracellular bacterial number at 0 time point between the wild-type, MAV_4644:Tn, and the complemented strain that is demonstrated by the colony formic units in the Figure 1, and by the percentage of invasion by the wildtype and the mutant MAV_4644:Tn strains in the Figure 6A for RAW264.7 macrophages. In addition, to validate whether MAV_4644 protein is relevant in human pathogenesis, in vitro infection assays were performed in THP-1 human macrophages.

We have to highlight that time 0 in our infection model is about 4 h post infection, which includes two hours of bacterial internalization to the host cells and then two hours of antibiotic treatment necessary to clear any extracellular bacteria. It was hypothesized that this observed difference (Figure 6A) is more likely a reflection of rapid killing by the macrophage instead of an impaired ability by MAV_4644:Tn to be phagocytosed by the host cell. To determine whether this was indeed the case, short-term infections were run in THP-1 cells (Figure 6B). The mycobacteria were allowed to invade for 0.5 h and cfu/ml were assayed up to 4 h. At 0.5 h post infection MAC104 and MAV_4644:Tn had been taken up at comparable levels. However, by 1 h post infection, about 60% of MAV_4644:Tn had been cleared by the host cells, while the MAC104 intracellular population remained constant throughout the duration of the experiment (Figure 6B). These data suggest the function of MAV_4644 protein serves to protect MAH from rapid killing in host macrophage and is not involved in invasion of macrophages.

### 3.9. MAV_4644 Interferes with the Cathepsin Z Function

**Figure 7:**
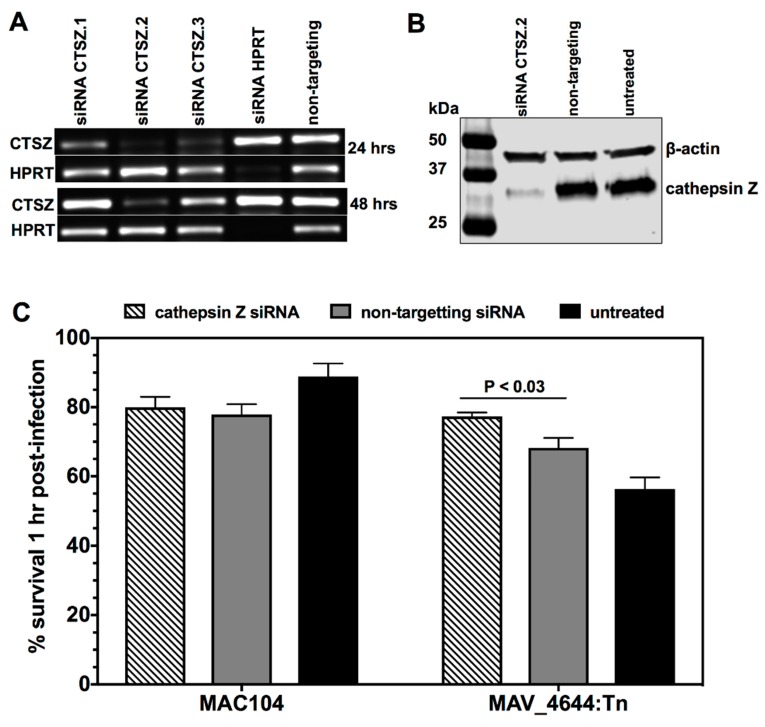
(**A**) Comparison of the CTSZ gene expression levels in THP-1 macrophages. The values are expressed as the number of cDNA copies detected by quantitative RT-PCR in samples obtained from 24 and 48 h transfected macrophages with Viromer^®^ GREEN transfection reagent (Lipocalyx). Three different CTSZ-targeting siRNAs were tested separately to identify the most effective targeting siRNA. The housekeeping gene hypoxanthine-guanine phosphoribosyltransferase (HPRT) transfection served as the positive control and non-targeting siRNA as the negative control. (**B**) The Western blot analysis of cathepsin Z protein in THP-1 macrophages at 4 days post transfection and at time 0 of infection. (**C**) One-hour survival assay of MAC104 and MAV_4644:Tn within CTSZ silenced and control macrophages. Data is reported as the average and standard error of two separate experiments. Survival of MAV_4644:Tn at 1 h post infection within cathepsin Z siRNA treated cells versus non-targeting siRNA treated cells was analyzed using Student’s *t* test. *, *p* < 0.05 was determined significant.

The cathepsin Z gene silencing of THP-1 macrophages was performed using Dicer-substrate short interfering RNAs. As the Figure 7A indicates, the cathepsin Z protein levels were significantly diminished at four days post transfection when compared to the negative control. Due to the fact that major killing of MAV_4644:Tn was observed at 1 h post infection of macrophages, we performed the survival assay at 1 h post infection in knock-down and control THP-1 cells. In cathepsin Z-silenced cells, MAV_4644:Tn survival rate was similar to the wild-type bacteria (Figure 7B). While MAC104 survival did not change in the knock-down control and was slightly more in untreated cells, MAV_4644:Tn colony forming units significantly decreased in both control groups (Figure 7B). Our data suggest that CTSZ is involved in early mycobacterial killing, and MAV-4644 protein plays role in suppressing of this process.

### 3.10. Effect of Cathepsin Z and NO on MAH

To determine whether cathepsin Z would have a direct bactericidal effect on bacterial killing in vitro, different MAH inoculums were exposed to up to 2 mM concentrations of CTSZ with or without the presence of 100 µmol of spermine NONOate, and bacterial CFUs were determined after 30 min incubation. As shown in the Table 4, while the cathepsin Z has no bactericidal effect on either the wild-type or MAV_4644:Tn clone, interestingly, the fast killing of knockout clone by cathepsin Z is only observed when a nitric oxide donor is added. Contrary, the wild-type MAC104 is resistant to CTSZ in the presence of NO.

## 4. Discussion

The mycobacterial response to nitric oxide within the host macrophage plays an important role in the survival and replication of these intracellular pathogens. MAH has been shown to require nitric oxide production by host cells for virulence in a murine model of infection [37]; however, an understanding of how the nitric oxide aids MAH success in intracellular survival is still unclear. Many bacterial pathogens, such as *P. aeruginosa*, *E. coli*, *S. aureus*, *S. typhimurium*, express nitrate, nitrite and nitric oxide reductases as virulence factors to metabolize these products into energy and to detoxify their niche [38]. Similar observations have been made with pathogenic mycobacteria, positing that the organisms are capable of nitrogen metabolism as an energy source and correlate with a positive effect on bacterial survival [39,40]. Furthermore, a bacterial genetics study proved that the nitrate reductase gene narG was required by MTB for intracellular growth. Nitric oxide serves as a potent signaling molecule and bactericidal effector within host macrophages and is required for intracellular growth of MAH. Nitric oxide could signal the macrophage to a more permissive state, serve as a virulence activator for the bacteria or be metabolized into energy. To investigate virulent-related genes of MAH associated with the resistance to oxidative killing mechanism of macrophages, we screened a transposon library and identified MAV_4644:Tn nitric oxide susceptible clone also attenuated in growth within host macrophages.

MAV_4644 is a putative ADP-ribosyltransferase (ADPRT), which is highly conserved throughout pathogenic mycobacteria and homologous to *M. tuberculosis* dual function channel protein with necrosis inducing toxin, Cpnt (Rv3903c). The homologous N-terminal domains (NTDs) are highly conserved in their primary sequences and the NTD of Cpnt has been shown to be a channel protein required for the uptake of glycerol [34,35]. MAV_4644:Tn displayed similar growth kinetics to wild-type MAH in media with glycerol as the sole carbon source, which could indicate a different function for this protein in MAH. However, a search for paralogous genes to MAV_4644 in the genome of MAC104 revealed MAV_2415, which could be a channel protein for glycerol uptake. There are no paralogous genes to Rv3903c in the genome of *M. tuberculosis* H37Rv. These findings could explain the differing phenotypes in the face of obvious identity. Interestingly, MAV_2415 does not share operonic conservation with MAV_4644 or Rv3903c.

The CTD of MAV_4644 is an ADPRT of the VIP2 super family. This family consists of toxins of pathogenic bacteria such as, *Pseudomonas aeruginosa* exoenzyme S (ExoS), *Vibrio cholerae* cholera toxin, *Bordetella pertussis* pertussis toxin, and *Corynebacterium diphtheriae* diphtheria toxin. In general, these proteins transfer ADP-ribose from NAD to host proteins, halting key cellular processes and intoxicating the host cell [26]. A pairwise alignment analysis in pBLAST of the primary protein sequences between the VIP2 domains of MAV_4644 and other known toxins revealed considerable conservation between MAV_4644 and ExoS of *P. aeruginosa*, as they aligned over 92% of the domain with an E-value of 2 × 10^−16^. In addition, Fieldhouse et al. identified MAV_4644 as an ExoS-like toxin utilizing computational tools to discover novel ADPRT toxins in bacterial pathogens [24].

ExoS is a type III secretion effector with a GTPase activating domain and an ADPRT domain required for *P. aeruginosa* virulence [41,42]. ExoS ADP-ribosylates Rab4 plays role in membrane trafficking and greatly inhibits the transferrin recycling [41]. ExoS also intoxicates cells by ribosylating Rab5 with ADP and interrupts the endosome fusion [43]. It has also been shown that the ADPRT activity of ExoS is required for *P. aeruginosa* to disrupt the pulmonary vascular barrier during pneumonic infection and to disseminate into the blood [44]. Interestingly, there is evidence for this family of toxins to spread between bacterial species by horizontal gene transfer [45]. Many ADPRT genes are encoded by mobile genetic elements such as bacteriophages, pathogenicity islands and plasmids, enabling promiscuity of this gene family [46]. In fact, the ADPRT domain of MAV_4644 is 53% GC, while the rest of the gene is 74%, which could be explained by horizontal gene transfer.

MAV_4644 is in an operon with MAV_4643 and MAV_4642, which are Esat-6 and CFP-10-like, and homologs of MTB esxE and esxF, respectively. Esat-6 and CFP-10 are a pair of sequential effector proteins secreted by the ESX-1 type VII secretion system of *M. tuberculosis* and are required for virulence [21,47,48,49]. All other Esat-6 and CFP-10-like gene pairs in both MAH and *M. tuberculosis* are associated with a gene clusters in ESX type VII secretion systems [33]. The fact that MAV_4643, MAV_4642 and their homologs esxE and esxF are upstream of a channel forming protein adds considerable weight to the MAV_4644 operon to be a secretion system of mycobacteria.

MAV_4644:Tn is attenuated in macrophages and displayed a rapid decline in the intracellular population in the first hour following macrophage infection. The function of MAV_4644 protein and/or operonic partners appears to protect the bacteria from early macrophage killing. MAV_4644_CTD, MAV_4643 and MAV_4642 recombinant proteins interact with cathepsin Z in an immunoprecipitation assay, which could indicate that all three proteins function together in complex to interfere with the host protein function.

**Figure 8:**
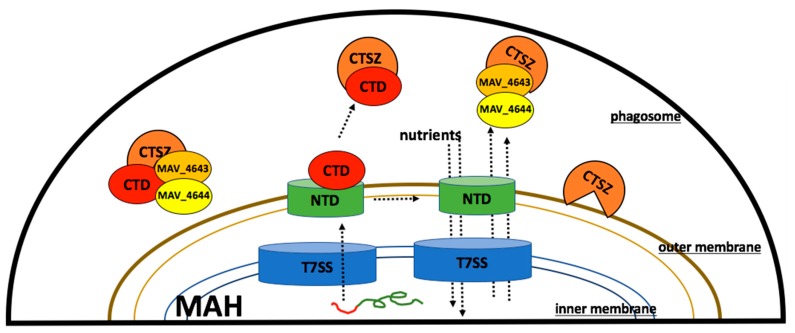
Hypothetical model of MAV_4644 protein interaction with cathepsin Z in the macrophage phagosome. MAV_4644 protein is transported via T7SS and assembles on the outer membrane. CTD is secreted through proteolytic cleavage, allowing CTD to interact with CTSZ, and opening NTD to transport nutrients and proteins. T7SS: type 7 secretion system, NTD: MAV_4644 protein N-terminal domain, CTD: MAV_4644 protein C-terminal domain, CTSZ: cathepsin Z protein.

Cathepsins are lysosomal peptidases, which act as important regulators of inflammation and cell death within macrophages [50]. There are 11 human cysteine cathepsins and many are involved in pathogenic mycobacterial infections [14,51]. Infection with MAH and *M. tuberculosis* inhibits the maturation of cathepsin L, while infection with *M. bovis* BCG inhibits expression of cathepsin S [52,53]. Most importantly, independent studies have associated single nucleotide polymorphisms in Cathepsin Z with increased susceptibility to tuberculosis [54,55]. CTSZ is a lysosomal cysteine peptidase [56]. It has been identified as a protein of the early endosome and such proteins are accessible to the mycobacterial vacuole [12,57]. Cathepsin Z has also been reported to be critical for degrading polyglutamine-containing proteins within lysosomes. The cell membranes of pathogenic mycobacteria have numerous polyglutamine stretches [58,59,60]. We propose that CTSZ possibly acts on the mycobacterial membrane early in infection, killing a subset of the phagocytosed bacteria (Figure 8). The secreted gene products of the MAV_4644 operon may serve to protect MAH from the peptidase, explaining the attenuation of the MAV_4644:Tn mutant. In the proposed model, MAV_4644 could ADP-ribosylate and inactivate cathepsin Z or MAV_4643 protein and MAV_4642 protein might interact with the active site or induce allosteric changes through protein-protein interactions.

To investigate this hypothesis, the CTSZ gene was silenced in THP-1 macrophages, and subsequent infection with MAV_4644:Tn and MAC104 were monitored. We could demonstrate that the knocking-down of cathepsin Z rescues the attenuation phenotype of MAV_4644:Tn to near wild-type levels of survival. The data suggest that CTSZ is involved in early mycobacterial killing of host macrophages and virulence factor MAV_4644 protein abrogates this process. Moreover, an evaluation of recombinant cathepsin Z shows that the protein exhibits the anti-MAH effect in conjunction with nitric oxide, even when the bactericidal activity is evaluated very early (only at 30 min). Our finding advances the knowledge on MAH pathogenesis mechanisms used to survive the action of macrophages. This study identified MAV_4644 as a one of important virulence factors for MAH. In addition, we demonstrated that the lysosomal peptidase cathepsin Z is involved in the early macrophage killing of mycobacteria by facilitating the function of NO. Further studies may identify the molecular relationship between cathepsin Z and NO controlling the macrophage killing method.

## Figures and Tables

**Table 1:** The list of the nitric oxide susceptible *Mycobacterium avium* subspecies *hominissuis* (MAH) mutants. MmT7 knockout clones were identified as those with 50% or less optical density when compared to the wild-type MAH after 48 h of growth in 7H9 broth supplemented with 10% OADC and 100 µM spermine NONOate. MmT7 transposon insertion sites were identified by LM-PCR and Sanger sequencing.

Gene ID	Clone	% of WT	Description	Uniprot Accession	Locus Tag
MAV_0118	2B2	16.2	Ppe family protein Subtilase family protein Monooxygenase, flavin-binding family protein Uncharacterized protein Uncharacterized protein Short chain dehydrogenase/reductase family protein Uncharacterized protein 3-Hydroxyacyl-CoA dehydrogenase type-2 Uncharacterized protein Gp108 protein RNA polymerase sigma factor Aldehyde dehydrogenase (NAD) family protein Putative acyl-CoA dehydrogenase Amidohydrolase 2 Uncharacterized protein Acyl-CoA dehydrogenase, C-domain protein Linear gramicidin synthetase subunit D Glycogen debranching enzyme GlgX Uncharacterized protein Uncharacterized protein Monooxygenase GTP pyrophosphokinase 2′-Hydroxybiphenyl-2-sulfinate desulfinase Ppe family protein Dehydrogenase 3-Ketosteroid dehydrogenase Isocitrate dehydrogenase, NADP-dependent Putative methyl transferase Putative NAD(+) arginine ADP-ribosyltransferase Cyclopropane-fatty-acyl-phospholipid synthase 2 Dehydrogenase Short chain dehydrogenase/reductase family protein NAD(P) transhydrogenase beta subunit Short chain dehydrogenase/reductase family protein	A0A0H2ZRQ5	MAV_RS00580
MAV_0158	1D3	40.1		A0A0H2ZRQ5	MAV_RS00770
MAV_0175	2D10	33.2		A0A0H2ZWF6	MAV_RS00840
MAV_0249	2F1	33.7		A0A0H2ZY15	MAV_RS01200
MAV_0273	1A9	39.7		A0A0H2ZWS7	MAV_RS01315
MAV_1264	1 E10	2.3		A0A0H2ZTF8	MAV_RS06065
MAV_1621	1G5	42.0		A0A0H2ZZA4	MAV_RS07760
MAV_1812	2F10	31.9		A0A0H3A4S5	MAV_RS08680
MAV_1816	1 E8	13.2		A0A0H2ZUN3	MAV_RS08700
MAV_2256	2D12	25.1		A0A0H3A3J1	MAV_RS10765
MAV_2426	1B12	1.5		A0A0H2ZZS1	MAV_RS11565
MAV_2564	1F10	6.8		A0A0H2ZWS2	MAV_RS12255
MAV_2590	2H4	33.9		A0A0H3A2H6	MAV_RS12365
MAV_2591	1H11	6.5		A0A0H2ZVD5	MAV_RS12360
MAV_2686	1B4	43.9		A0A0H2ZUN8	MAV_RS12825
MAV_2767	1 E3	40.1		A0A0H3A0C8	MAV_RS13195
MAV_3056	1B6	39.4		A0A0H2ZTY5	MAV_RS14575
MAV_3210	1H4	34.8		A0A0H2ZZU9	MAV_RS19660
MAV_3286	2F7	36.0		A0A0H2ZX00	MAV_RS15695
MAV_3296	2F3	43.5		A0A0H2ZRF2	MAV_RS15740
MAV_3337	1F1	11.1		A0A0H2ZWN3	MAV_RS15945
MAV_3464	2F6	29.9		A0A0H3A3N2	MAV_RS16570
MAV_3808	2 E11	38.2		A0A0H3A3M7	MAV_RS18245
MAV_4014	2A4	39.1		A0A0H2ZTW6	MAV_RS19230
MAV_4124	2F12	27.4		A0A0H2ZX25	MAV_RS19785
MAV_4249	2D11	41.6		A0A0H3A167	MAV_RS20390
MAV_4313	1 E12	0.9		A0A0H2ZT90	MAV_RS20695
MAV_4573	1G2	34.6		A0QLB4	MAV_RS21960
MAV_4644	1F2	29.0		A0QLI5	MAV_RS22305
MAV_4647	2G2	38.2		A0A0H2ZZ17	MAV_RS22320
MAV_4791	1D5	34.1		A0A0H2ZUD0	MAV_RS23020
MAV_4916	1A11	22.1		A0A0H3A2P2	MAV_RS23625
MAV_5140	1D2	40.9		A0A0H2ZXD2	MAV_RS24655
MAV_5205	2F8	38.4		A0A0H3A044	MAV_RS24970

**Table 2:** Conserved domain analysis of MAV_4644 protein.

Name	Accession	Description	Amino Acids	E-Value
WXG100	Pfam06013	Proteins of 100 residues with WXG	7–92	0.00371
DedD	COG3147	Cell division protein DedD	455–527	0.00631
PHA03247	PHA03247	Large tegument protein UL36	455–655	5.69 × 10^−9^
Atrophin-1	Pfam03154	Atrophin-1 family	459–634	2.74 × 10^−6^
Rad23	TIGR00601	UV excision repair protein Rad23	475–543	0.00525
ADPrib_exo_Tox	Pfam03496	ADP-ribosyltransferase exoenzyme	675–822	4.03 × 10^−15^
VIP2	Cd00233	Family of actin-ADP-ribosylating toxin	675–820	1.18 × 10^−14^

**Table 3:** The MAV_4644:Tn complemented clone survival in Raw 264.7 macrophages.

Experimental Groups	Infection CFU/mL (Time 0)	Infection CFU/mL (Time 4 Days)
Wild-type MAH	6.1 ± 0.5 × 10^5^	7.8 ± 0.4 × 10^5^
MAV_4644:Tn	2.6 ± 0.3 × 10^5^	1.3 ± 0.3 × 10^4^ *
Complemented MAV_4644:Tn	5.8 ± 0.3 × 10^5^	6.9 ± 0.5 × 10^5^

*, *p* < 0.05 compared with CFUs/mL of the wild-type MAH and the complemented clone. Mean ± SD of four independent experiments. Infection occurred for 1 h. Inoculum for the three strains, MAC104, MAV_4644:Tn and complemented strains, was 1 ± 0.4 × 10^6^.

**Table 4:** In vitro effects of the recombinant cathepsin Z protein on MAH with or without nitric oxide presence.

Cathepsin Z (mM/mL)	MAC104/MAV_4644:Tn CFU/mL		
	10^3^	10^4^	10^5^
0	1.3 × 10^3^/1.1 × 10^3^	1.2 × 10^4^/1.2 × 10^4^	1.3 × 10^5^/1.2 × 10^5^
0 + NO	1.4 × 10^3^/9.1 × 10^2^	1.4 × 10^4^/8.3 × 10^3^	1.1 × 10^5^/8.1 × 10^4^
0.5	1.3 × 10^3^/ 1.4 × 10^3^	1.5 × 10^4^/ 1.2 × 10^4^	1.4 × 10^5^/1.3 × 10^5^
0.5 + NO	1.3 × 10^3^/ 7.2 × 10^2^ *	1.3 × 10^4^/6.4 × 10^3^ *	1.3 × 10^5^/7.8 × 10^4^ *
1.0	1.4 × 10^3^/1.3 × 10^3^	1.4 × 10^4^/1.2 × 10^4^	1.4 × 10^5^/1.2 × 10^5^
1.0 + NO	1.3 × 10^3^/5.3 × 10^2^ *	1.5 × 10^4^/4.2 × 10^3^ *	1.4 × 10^5^/4.7 × 10^4^ *
2.0	1.4 × 10^3^/1.2 × 10^3^	1.3 × 10^4^/1.2 × 10^4^	1.2 × 10^5^/1.1 × 10^5^
2.0 + NO	1.2 × 10^3^/1.8 × 10^2^ *	1.3 × 10^4^/1.4 × 10^3^ *	1.3 × 10^5^/9.9 × 10^3^ *

* is <0.05 compared with Cathepsin Z without NO.

## Supplementary Materials

The supplementary materials are available online at https://www.mdpi.com/2076-2607/7/5/144/s1.
