# Supplementary material for: MAV_4644 Interaction with the Host Cathepsin Z Protects Mycobacterium avium subsp. hominissuis from Rapid Macrophage Killing
Source: Microorganisms. 2019 May 21;7(5):144. doi: 10.3390/microorganisms7050144 (PMC6560410; doi:10.3390/microorganisms7050144)
Supplement: Supplementary file 1 [file microorganisms-07-00144-s001.pdf]

Supplemental table S1. Immunoprecipitation of THP-1 protein by MAV_4644_CTD, MAV_4643, and MAV_4642 recombinant proteins. MAH proteins were produced using traditional cloning methods and captured on His-60 Ni Superflow gravity columns. Host lysate was added to the columns and incubated over night at 4°C with gentle inversion. The columns were washed and then bound proteins were eluted. The proteins were sequenced using LC-MS MS at the Oregon State University Mass Spectrometry Center.

MAV_4644_CTD protein

Accession	Description	MW [kDa]
Q5U000	Cathepsin Z	33.8
A8K9C4	Elongation factor 1-alpha	50.2
B3KUJ0	Splicing factor 3B subunit	44.5

MAV_4643 protein

Accession	Description	MW [kDa]
Q9H4X1	Regulator of cell cycle	14.6
Q5U000	RGCC Cathepsin Z	33.8
A0A0K2BMD	Mutant hemoglobin alpha 2 globin chain	15.2
8	complement component 1, q subcomponent binding protein	31.4
A8K651	(C1QBP) Transmembrane glycoprotein NMB	68
B4E3D4	GRB2-related adapter	22.1
I3L2P9	protein cadherin 4, type 1,	100.2
[illegible]	[illegible]	

MAV_4642 protein

Accession	Description	MW [kDa]
Q5U000	Cathepsin Z	33.8
A6XMH4	Beta-2-microglobulin	14.4
A0A024RBH	Cytoskeleton-associated protein 4, isoform	66
2	CRA_c Integrin beta	83.6
Q6PJ75	Elongation factor 1-alpha	50.2
A8K9C4	MHC class I antigen	53.8
A0A060CZ20	Lysosome-associated membrane glycoprotein 1	44.9
P11279	nucleobindin 1 (NUCB1)	23.9
A8K7Q1	Heterogeneous nuclear ribonucleoprotein H	51.2
G8JLB6	Macrophage scavenger receptor types I	49.7
P21757	and II Adenylyl cyclase-associated protein	51.6
D3DPU2	MHC class I antigen	40.4
A0A024F8H3	scaffold attachment factor B (SAFB)	102.7
A8K329	Scavenger receptor cysteine-rich type 1 protein	125.4
Q86VB7	M130 HSPA9 protein	73.8
Q8N1C8	serpin peptidase inhibitor, clade H (heat shock protein 47), member 1	46.5
A8K259	ADP-dependent	54.1
Q9BRR6	glucokinase EBNA-2 co-	107.4
Q59FF0	activator variant Cathepsin	37.5
P25774	S	58.3
Q9HAT2	Sialate O-acetylesterase	68
B4E3D4	Transmembrane glycoprotein	69.3
B2RE46	NMB ribophorin II (RPN2)	54.7
A0A024R0Q	Phospholipase D family, member 3, isoform CRA_b	42
4	actin, alpha, cardiac muscle (ACTC)	123.9
A8K3K1	Family with sequence similarity 62 (C2 domain	251.7
A0A024RB16	containing) Protein transport protein Sec16A	136.1
J3KNL6	SWI/SNF complex subunit	112.9
F8VXC8	SMARCC2 Neutral alpha-	50.7
B4DJ30	glucosidase AB	
A0A024RAD	Dolichyl-diphosphooligosaccharide--protein glycosyltransferase	

continued:

P05362	Intercellular adhesion molecule 1	57.8
Q13435	Splicing factor 3B subunit 2	100.2
Q53HV2	Chaperonin containing TCP1, subunit 7 (Eta) variant	59.3
A0A087WSV	Nucleobindin 2, isoform CRA_b	50.2
B2R6S9	low density lipoprotein receptor-related protein associated protein 1	41.5
Q58EY4	SWI/SNF related, matrix associated, actin dependent regulator of	122.8
B2R7P8	5-aminoimidazole-4-carboxamide ribonucleotide formyltransferase	64.6
B5BUE6	ATP-dependent RNA helicase DDX5	69.1
Q5VZU9	Tripeptidyl-peptidase 2	139.7
Q9P0K7	Ankycorbin	110
B7Z6Z4	Myosin light polypeptide 6	26.7
B7Z507	Matrix metalloproteinase-9	71.5
Q9HCC0	Methylcrotonoyl-CoA carboxylase beta chain, mitochondrial	61.3
P09382	Galectin-1	14.7
Q6ICQ8	ARHG protein	21.3
Q8NCA5	Protein FAM98A	55.4
B2RDQ3	splicing factor, arginine/serine-rich 10	33.7
Q06830	Peroxiredoxin-1	22.1
A6NC86	phospholipase A2 inhibitor and Ly6/PLAUR domain-containing protein	21.9
B8XPJ8	Membrane bound catechol-O-methyltransferase	30
A0A024QZY	PRP4 pre-mRNA processing factor 4 homolog B	116.9
J3KPS3	Fructose-bisphosphate aldolase	39.8
P39023	60S ribosomal protein L3	46.1
A0A024R5X3	SAFB-like, transcription modulator, isoform CRA_b	131.5
B7ZLW0	LPP protein	65.7
P23526	Adenosylhomocysteinase	47.7
A0A0J9YXJ0	CUGBP Elav-like family member 2	57.1
A8K517	Ribosomal protein S23, isoform CRA_a	15.8
E9PRY8	Elongation factor 1-delta	76.5
Q4LE40	C14orf159 variant protein	68
A8MUS3	60S ribosomal protein L23a	21.9
Q59H77	T-complex protein 1 subunit gamma	63.5
P26641	Elongation factor 1-gamma	50.1
E7D7X9	Pyrroline-5-carboxylate reductase	33.4
P50990	T-complex protein 1 subunit theta	59.6
F8VU51	YLP motif-containing protein 1	160.6
Q1HBJ4	Mitogen-activated protein kinase	41.4
B7Z4C8	60S ribosomal protein L31	15.1
Q8WUA2	Peptidyl-prolyl cis-trans isomerase-like 4	57.2
A8K6V3	splicing factor 3b, subunit 3, 130kDa (SF3B3)	135.5
P62701	40S ribosomal protein S4, X isoform	29.6
P23219	Prostaglandin G/H synthase 1	68.6
A0A0S2Z5U3	Heterogeneous nuclear ribonucleoprotein L-like isoform 2	63.6
Q96ST3	Paired amphipathic helix protein Sin3a	145.1
B2R4C0	60S ribosomal protein L18a	20.7
P62633	Cellular nucleic acid-binding protein	19.4
Q7L2J0	7SK snRNA methylphosphate capping enzyme	74.3
B4DNE1	Basigin	42.2
G3V2S9	SRA stem-loop-interacting RNA-binding protein, mitochondrial	13.9
Q13595	Transformer-2 protein homolog alpha	32.7
A0A0A6YYJ8	Putative RNA-binding protein Luc7-like 2	54.2
Q9Y4E8	Ubiquitin carboxyl-terminal hydrolase 15	112.3
P11215	Integrin alpha-M	51

continued:

Q8IVS2	Malonyl-CoA-acyl carrier protein transacylase, mitochondrial	42.9
Q9NYF8	Bcl-2-associated transcription factor 1	37.5
A0A0D9SGE	PHD finger protein 6	41.3
Q9H0N5	Pterin-4-alpha-carbinolamine dehydratase 2	14.4
P23368	NAD-dependent malic enzyme, mitochondrial	22.8
Q6U8A4	Ubiquitin-specific protease 7 isoform	128.9
O43290	U4/U6.U5 tri-snRNP-associated protein 1	90.2
A0A0S2Z570	Retinoid X receptor beta isoform 2	57.3
A0A024R845	RAB14, member RAS oncogene family, isoform CRA_a	40.9
A0A024R5Q	Glycine amidinotransferase (L-arginine:glycine amidinotransferase)	54.1
Q53Z07	NPC-A-16	21.8
O15294	UDP-N-acetylglucosamine--peptide N-acetylglucosaminyltransferase	116.8
A0A0C4DG1	40S ribosomal protein SA	33.3
B4DJQ5	Glucosidase 2 subunit beta	60.1
P54886	Delta-1-pyrroline-5-carboxylate synthase	87.2
Q53FI7	Four and a half LIM domains 1 variant (Fragment)	31.9
O14874	[3-methyl-2-oxobutanoate dehydrogenase [lipoamide]] kinase	46.3
A0A090KM5	HLA class I antigen	40.3
B2R6P1	galactosamine (N-acetyl)-6-sulfate sulfatase	57.9
Q8TD55	Pleckstrin homology domain-containing family O member 2	53.3
P62979	Ubiquitin-40S ribosomal protein S27a	18
A0A0A0MT8	Isovaleryl-CoA dehydrogenase, mitochondrial	46.6
A0A024QZN	Voltage-dependent anion channel 2, isoform CRA_a	34.5
E7EMB3	Calmodulin	21.7
Q9BWJ5	Splicing factor 3B subunit 5	10.1
Q59GK9	Ribosomal protein L21 variant (Fragment)	18.9
A0A024R608	Ribosomal protein, large, P1, isoform CRA_a	11.5
Q9NW64	Pre-mRNA-splicing factor RBM22	46.9
P20674	Cytochrome c oxidase subunit 5A, mitochondrial	16.8
O43660	Pleiotropic regulator 1	57.2
Q9C0J8	pre-mRNA 3' end processing protein WDR33	145.8
Q9BV19	Uncharacterized protein C1orf50	21.9
Q05DF2	SF3A2 protein	51.4
Q9Y2W1	Thyroid hormone receptor-associated protein 3	108.6
P52948	Nuclear pore complex protein Nup98-Nup96	197.5
A0A0U1RRM	Polypyrimidine tract-binding protein 1	62.4
B4E141	golgi SNAP receptor complex member 2	26.9
Q13405	39S ribosomal protein L49, mitochondrial	19.2
Q14241	Transcription elongation factor B polypeptide 3	89.9
Q9Y6M5	Zinc transporter 1	55.3
Q59EI9	ADP,ATP carrier protein, liver isoform T2 variant	35.4
Q9Y2X9	Zinc finger protein 281	96.9
Q13151	Heterogeneous nuclear ribonucleoprotein A0	30.8
B2R9K8	chaperonin containing TCP1, subunit 6A (zeta 1)(CCT6A)	57.9
P14317	Hematopoietic lineage cell-specific protein	54
B4DIV8	Tripeptidyl-peptidase 1 (EC 3.4.14.9)	62.2
J3QK89	Calcium homeostasis endoplasmic reticulum protein	104.9
Q16629	Serine/arginine-rich splicing factor 7	27.4
Q6PJT7	Zinc finger CCCH domain-containing protein 14	82.8
Q6IPI1	Ribosomal protein L29	17.9
A0A024RDE	PDZ and LIM domain 5, isoform CRA_c	63.9
Q15717	ELAV-like protein 1	36.1
P56270	Myc-associated zinc finger protein	48.6
